# Infiltrating immune cells in benign breast disease and risk of subsequent invasive breast cancer

**DOI:** 10.1186/s13058-021-01395-x

**Published:** 2021-01-30

**Authors:** Thomas E. Rohan, Rhonda Arthur, Yihong Wang, Sheila Weinmann, Mindy Ginsberg, Sherene Loi, Roberto Salgado

**Affiliations:** 1 Department of Epidemiology and Population Health, Albert Einstein College of Medicine, 1300 Morris Park Ave., Bronx, NY 10461 USA; 2 Department of Pathology and Laboratory Medicine, Rhode Island Hospital and Lifespan Medical Center, Warren Alpert Medical School of Brown University, Providence, RI 02903 USA; 3 Center for Health Research, Kaiser Permanente Northwest, Portland, OR USA; 4 Sir Peter MacCallum Department of Oncology, University of Melbourne, Parkville, Australia; 5 Division of Research, Peter MacCallum Cancer Centre, Melbourne, Australia; 6 Department of Pathology, GZA-ZNA, Antwerp, Belgium

**Keywords:** Tumor-infiltrating lymphocytes, Benign breast disease, Breast cancer

## Abstract

**Background:**

It is well established that tumors are antigenic and can induce an immune response by the host, entailing lymphocytic infiltration of the tumor and surrounding stroma. The extent and composition of the immune response to the tumor, assessed through evaluation of tumor-infiltrating lymphocyte counts, has been shown in many studies to have prognostic and predictive value for invasive breast cancer, but currently, there is little evidence regarding the association between infiltrating immune cell counts (IICCs) in women with benign breast disease (BBD) and risk of subsequent invasive breast cancer.

**Methods:**

Using a cohort of 15,395 women biopsied for BBD at Kaiser Permanente Northwest, we conducted a nested case-control study in which cases were women who developed a subsequent invasive breast cancer during follow-up and controls were individually matched to cases on age at BBD diagnosis. We assessed IICCs in normal tissue and in the BBD lesions, and we used unconditional logistic regression to estimate the multivariable odds ratios (OR) and 95% confidence intervals (CI) for the associations between IICCs and breast cancer risk.

**Results:**

There was no association between the IICC in normal tissue (multivariable OR per 5% increase in IICC = 1.05, 95% CI = 0.96–1.16) or in the BBD lesion (OR per 5% increase in IICC = 1.06, 95% CI = 0.96–1.18) and risk of subsequent invasive breast cancer. Also, there were no associations within subgroups defined by menopausal status, BBD histology, BMI, and history of smoking.

**Conclusion:**

The results of this study suggest that IICCs in BBD tissue are not associated with altered risk of subsequent invasive breast cancer.

## Introduction

It is well established that tumors are antigenic and can induce an immune response by the host, entailing lymphocytic infiltration of the tumor and surrounding stroma [1]. The extent and composition of the immune response to the tumor, assessed through evaluation of tumor-infiltrating lymphocytes (TILs), has been shown in many studies to have prognostic and predictive value for invasive breast cancer, mostly for triple-negative breast cancer (TNBC) and the HER2+ subtype [2].

The recent observation that lower relative counts of cytotoxic CD8^+^ cells and higher relative counts of regulatory FOXP3^+^ T cells were associated with increased breast cancer risk [3] indicates that the effect of the host immune response on disease progression may be observable at a relatively early stage in the natural history of breast cancer. In this regard, women with benign breast disease (BBD) are of potential relevance, because despite their having an increased risk of subsequent invasive breast cancer [4], their BBD does not necessarily progress. This suggests that factors beyond BBD must influence the likelihood of progression, and in this regard, the immune contexture may be relevant.

Currently, little is known about the significance of the immune infiltrate in putative breast cancer precursors [1]. One recent study showed no association between TILs in ductal carcinoma in situ and risk of an ipsilateral breast event [5], while another suggested that reduced B-cell infiltration in BBD tissue was associated with increased risk of subsequent breast cancer [6].

Given the paucity of current evidence, in the study reported here, we examined the association between infiltrating immune cell counts (IICCs) in BBD tissue and risk of subsequent invasive breast cancer. Here, we used the term IICCs in preference to TILs because BBD lesions are considered to be “benign.”

## Materials and methods

### Study population

The study population and the study design have been described in detail elsewhere [7]. In brief, we conducted a case-control study nested within a cohort of 15,395 women who had a biopsy for BBD within the Kaiser Permanente Northwest Region (KPNW) health care system between 1971 and 2006 and were followed to mid-2015 for subsequent invasive breast cancer. Cases were the 526 women with a biopsy for BBD who developed a subsequent invasive breast cancer at least 1 year after the index BBD biopsy, and controls (1/case; *n* = 526) were women with a biopsy for BBD who were alive but had not developed breast cancer during the same follow-up period as that for the corresponding case. Risk factor data were abstracted from the KPNW medical records.

### Histopathology

Hematoxylin and eosin sections were prepared from BBD tissue blocks and reviewed according to standard pathologic criteria as defined in the original study [7]. Lesions were defined as follows: no lesion/non-proliferative lesion (cysts, fibrosis, apocrine metaplasia, adenosis, simple fibroadenoma); proliferative disease without atypia (usual ductal hyperplasia; columnar cell change and columnar cell hyperplasia; complex fibroadenoma; sclerosing adenosis; radial scar; complex sclerosing lesion, papilloma); and proliferative disease with atypia (atypical ductal hyperplasia, atypical lobular hyperplasia, columnar cell lesion with atypia, including flat epithelial atypia). Each biopsy specimen was further categorized for presence or absence of lobular involution. Since the distinction between a proliferative lesion with and without atypia is poorly reproducible between pathologists without using immunohistochemistry [8], the original diagnosis of all lesions in the underlying study [7] was used. In breast sections with a mix of lesions, such as (atypical) ductal hyperplasia surrounded by papillomas and regions with columnar cell change and sometimes a fibroadenoma, the IICCs were scored across all lesions.

### Infiltrating immune cell counts

IICCs were assessed using the same principles as defined for TILs by the International Immuno-Oncological Working group (www.tilsinbreastcancer.org), in a manner similar to that described previously for DCIS [5] and according to an established guideline for scoring TILs in DCIS [9]. This method has been demonstrated to be reproducible among pathologists [10]. The denominator used to determine the % stromal IICCs was the area of stromal tissue (i.e., area occupied by mononuclear inflammatory cells over total perilesional stromal area surrounding each lesion, not the number of stromal cells (i.e., fraction of total stromal nuclei that represent mononuclear inflammatory cell nuclei)). The lymphocytes were scored in normal lobules and in lesions.

### Statistical analysis

Multivariable odds ratios (OR) and 95% confidence intervals (CI) for the associations of IICCs (examined both as continuous (per 5% increase) and as categorical variables (< 5%, ≥ 5%)) in normal tissue and in the BBD lesions with risk of invasive breast cancer were estimated using unconditional logistic regression with adjustment for the following variables: age at enrollment, body mass index (BMI), menopausal status, ever use of hormonal therapy, family history of breast cancer, ever smoked cigarettes, pack-years of cigarette smoking, age at menarche, age at first birth/parity, and BBD histology (unless included as the main exposure). For analysis purposes, the BBD lesions were categorized as non-proliferative or proliferative (with or without atypia).

## Results

**Fig. 1:**
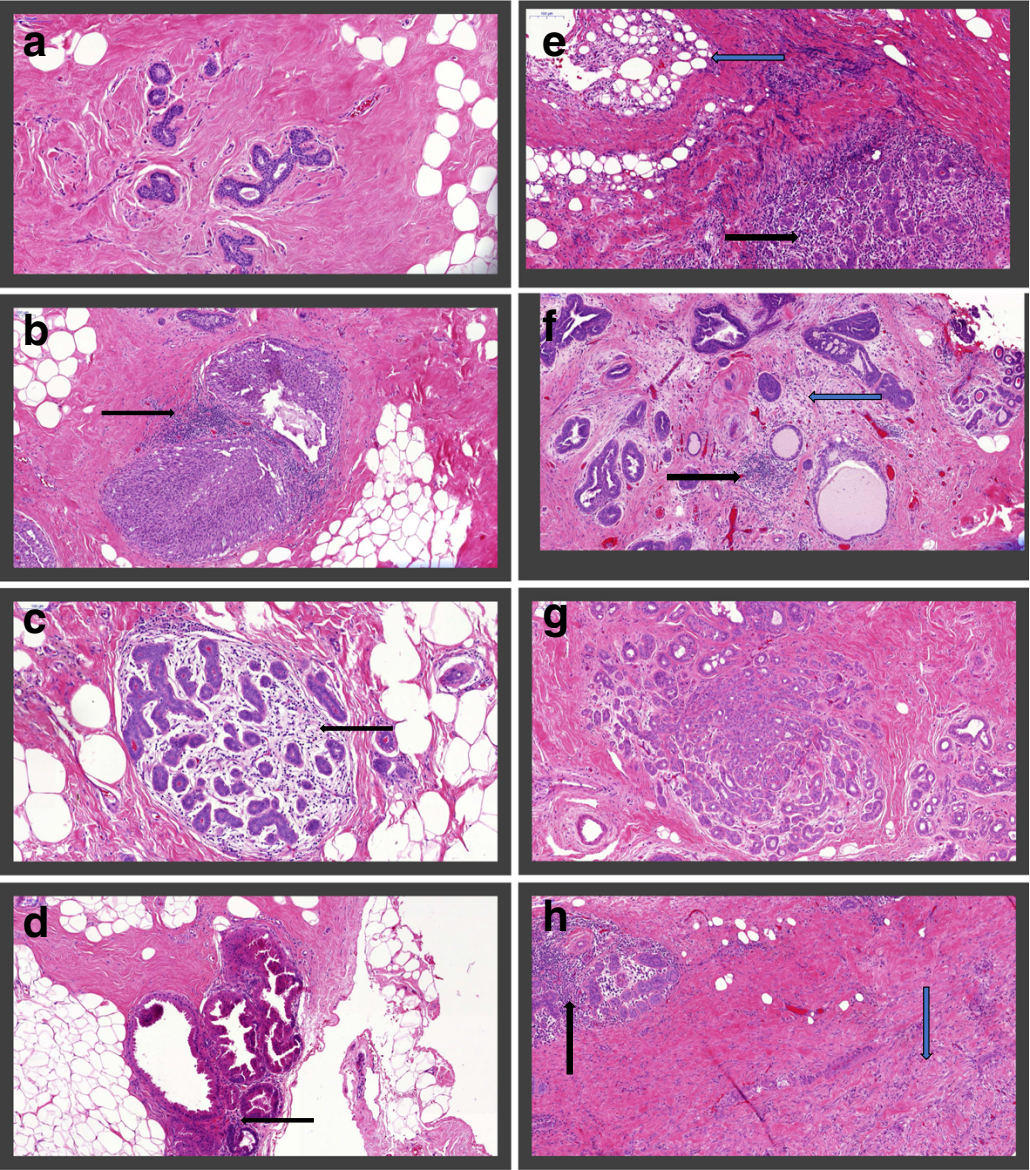
**a** Normal ducts without infiltrating immune cells. **b** Usual ductal hyperplasia with infiltrating immune cells (black arrow). **c** Normal lobule with infiltrating immune cells (black arrow). **d** Apocrine metaplasia with minimal infiltrating immune cells (black arrow). **e** Normal lobule with many infiltrating immune cells (black arrow), near a zone of scarring (blue arrow). **f** Usual ductal hyperplasia, ductal cysts, and infiltrating immune cells (black arrow) in areas of stromal remodeling (blue arrow). **g**: Sclerosing adenosis without infiltrating immune cells. **h** Normal lobule with infiltrating immune cells (black arrow) near a zone of scarring (blue arrow)

Illustrative examples of IICCs in various lesions are shown in Fig. 1. The associations between IICCs in normal and BBD tissue and breast cancer risk are shown in Table 1. There was no association between the IICCs in normal tissue and risk of subsequent invasive breast cancer. When examined as a continuous variable, the adjusted odds ratio for the increase in risk per 5% increase in IICC was 1.05 (95% CI 0.96–1.16). Similarly, there was no association between the IICC in the BBD lesion and breast cancer risk (OR per 5% increase in IICC = 1.06, 95% CI 0.96–1.18). There were also no associations within subgroups defined by menopausal status, BBD histology, BMI, and history of cigarette smoking (Table 1).

**Table 1:** Association between infiltrating immune cell counts (IICCs) in women biopsied for benign breast disease and risk of subsequent invasive breast cancer risk

	IICCs in normal tissue (%)				IICCs in BBD lesion (%)			
	Per 5% increase	< 5	≥ 5	Missing	Per 5% increase	< 5	≥ 5	Missing
**Overall**								
Cases/controls		424/422	84/81	11/17		326/335	93/82	100/103
Age-adjusted OR	1.05 (0.96–1.15)	1.00	1.04 (0.74–1.45)	–	1.04 (0.94–1.16)	1.00	1.16 (0.83–1.62)	–
Multivariable-adjusted OR^a^	1.05 (0.96–1.16)	1.00	1.04 (0.74–1.48)	–	1.06 (0.96–1.18)	1.00	1.11 (0.79–1.58)	–
**Menopausal status**								
**Premenopausal**								
Cases/controls		165/142	41/38	3/7		131/122	42/23	36/42
Age-adjusted OR	1.02 (0.91–1.13)	1.00	0.95 (0.58–1.56)	–	1.17 (0.97–1.41)	1.00	1.73 (0.98–3.05)	–
Multivariable-adjusted OR	1.01 (0.90–1.14)	1.00	0.95 (0.56–1.63)	–	1.16 (0.96–1.40)	1.00	1.52 (0.83–2.79)	–
**Postmenopausal**								
Cases/controls		178/206	32/29	4/9		132/156	37/46	45/42
Age-adjusted OR	1.37 (1.01–1.85)	1.00	1.28 (0.75–2.21)	–	0.96 (0.82–1.13)	1.00	0.94 (0.57–1.54)	–
Multivariable-adjusted OR	1.34 (0.99–1.83)	1.00	1.19 (0.67–2.09)	–	0.99 (0.83–1.19)	1.00	0.98 (0.58–1.66)	–
***P*** **for heterogeneity**^**b**^		0.56				0.23		
**BBD**								
**Non-proliferative**								
Cases/controls		59/78	11/17	2/4		27/46	8/10	37/43
Age-adjusted OR	1.03 (0.85–1.25)	1.00	1.00 (0.98–1.01)	–	1.07 (0.79–1.44)	1.00	1.40 (0.40–4.00)	–
Multivariable-adjusted OR^c^	1.12 (0.90–1.39)	1.00	1.15 (0.45–2.95)	–	1.15 (0.69–1.91)	1.00	1.43 (0.42–4.82)	–
**Proliferative**								
Cases/controls		356/329	68/62	9/12		298/284	85/71	50/48
Age-adjusted OR	1.06 (0.95–1.17)	1.00	1.00 (0.99–1.01)	–	1.05 (0.94–1.18)	1.00	1.15 (0.80–1.63)	–
Multivariable-adjusted OR	1.03 (0.93–1.15)	1.00	0.94 (0.64–1.39)	–	1.06 (0.95–1.19)	1.00	1.07 (0.74–1.55)	–
***P*** **for heterogeneity**^**b**^		0.88				0.83		
**BMI**								
**18.5–24.9 kg/m**^**2**^								
Cases/controls		170/166	36/31	2/6		128/119	41/34	39/50
Age-adjusted OR	1.02 (0.87–1.19)	1.00	1.10 (0.65–1.88)	–	1.03 (0.88–1.22)	1.00	1.13 (0.67–1.90)	–
Multivariable-adjusted OR	1.10 (0.93–1.30)	1.00	1.23 (0.70–2.16)	–	1.10 (0.92–1.33)	1.00	1.18 (0.67–2.07)	–
**≥ 25.0 kg/m**^**2**^								
Cases/controls		208/210	37/38	7/6		165/166	35/45	52/43
Age-adjusted OR	1.05 (0.92–1.20)	1.00	1.02 (0.62–1.68)	–	0.95 (0.83–1.10)	1.00	0.74 (0.67–1.22)	–
Multivariable-adjusted OR	1.00 (0.88–1.15)	1.00	0.91 (0.54–1.53)	–	1.00 (0.86–1.17)	1.00	0.68 (0.40–1.14)	–
***P*** **for heterogeneity**^**b**^		0.45				0.23		
**Cigarette smoking**								
**No**								
Cases/controls		149/171	35/31	3/4		113/128	34/32	40/46
Age-adjusted OR	1.03 (0.85–1.25)	1.00	1.40 (0.82–2.40)	–	0.95 (0.78–1.14)	1.00	1.15 (0.66–1.99)	–
Multivariable-adjusted OR	1.12 (0.90–1.39)	1.00	1.49 (0.85–2.61)	–	0.92 (0.76–1.13)	1.00	1.09 (0.61–1.97)	–
**Yes**								
Cases/controls		173/185	25/32	5/5		139/155	32/30	32/37
Age-adjusted OR	1.06 (0.95–1.17)	1.00	0.82 (0.47–1.45)	–	1.06 (0.92–1.22)	1.00	1.20 (0.69–2.08)	–
Multivariable-adjusted OR	1.03 (0.93–1.15)	1.00	0.73 (0.40–1.35)	–	1.13 (0.97–1.32)	1.00	1.29 (0.72–2.32)	–
***P*** **for heterogeneity**^**b**^		0.12				0.86		

^a^Adjusted for age at enrollment, BMI, menopausal status, hormonal therapy use ever, family history of breast cancer, smoking ever, pack-years of smoking, age at menarche, age at first birth/parity, and benign breast disease histology unless included as the main exposure

^b^Values were derived from multivariable models with the categorical exposures

## Discussion

The assessment of TILs in breast cancer has proven prognostic and predictive importance [11]. The advantages of TIL evaluation in H&E sections include technical ease and reproducibility, but a limitation is that it does not inform regarding the proportions of different lymphocyte populations or the functional status of the infiltrates [2]. The importance of infiltrating immune cells in BBD is largely unknown. We conducted a case-control study of breast cancer nested within a cohort of 15,395 women who had a biopsy for BBD and did not find any association between IICC and breast cancer risk. Study strengths include a defined population, a substantial sample size, and assessment of immune cell counts blinded to outcome. Study weaknesses include the limited power to perform analyses stratified by breast cancer risk factors, missing values for some covariates, and the fact that no revision with immunohistochemistry could be done to confirm the previously made histological diagnoses. In addition, it is well known that the phase of the menstrual cycle influences the extent of the immune infiltrate in normal breast tissues [12]. For the premenopausal subjects in this study, we did not have information on the phase of the menstrual cycle at the moment of the tissue sampling. This may have led to non-differential misclassification of the IICCs, with resulting bias in the odds ratio estimates. In future studies, information on the menstrual phase needs to be collected at the moment of biopsy when studying immune infiltrates in benign and probably also in non-benign (pre-invasive and invasive) breast disease.

It is remarkable that the distribution of the IICCs that we observed is similar to that seen in luminal hormone receptor-positive (HR+) breast cancer and invasive lobular HR+ breast cancer, with most of the cases investigated in this study having between 0 and 10% IICC, reflecting low recognition by immunity. The main genomic drivers of immunity, if any, in hyperplasia, papilloma, and fibroadenoma, are still unknown, and it would be of interest to compare genomic findings for BBD, HR+ pre-invasive, and HR+ invasive cancer. Furthermore, the neoantigen-load in these lesions is also unknown. Since the range of lymphocyte counts in these lesions is the same as in luminal and invasive lobular cancer, the neo-antigen load might be similar. However, it may be important to the understanding of how BBD induces immunity to understand why TILs in luminal and invasive lobular cancer do not associate with a better prognosis, compared to HER2+ and TNBC disease.

There is evidence that the immune system plays a role in mammary postnatal organogenesis [13]. Hence, a role of immune cells beyond simple protective immunity might be envisaged, not only in benign breast disease but also in pre-neoplastic and neoplastic lesions. In the present study, some cases had normal breast lobules with many lymphocytes, while others had no lymphocytes. It was remarkable that some cases had lymphocyte-rich lobules (= lobulitis) near zones of scarring or inflammation/abscesses, while in the same case at a distance the lobules had no lymphocytes. This suggests that lymphocytes home in on lobules near areas of active stromal remodeling and inflammation. This pattern is also seen in DCIS and in invasive cancer (personal observation, RS). To date, there has been little research on the role of normal lobules in cancer immunity, but these findings suggest that lobules might have a role in lymphocyte maturation, in a manner similar to that of tertiary lymphoid structures (TLS). In diabetes, a higher proportion of patients have lobulitis, suggesting that in auto-immune disease and any other disease involving immunity, this may be reflected in pre-existing normal lobules. Also, higher levels of “lymphocytic lobulitis” around TNBC than in non-TNBC have been described [14]. A role of lobules in TIL-homing and maybe maturity is also suggested by the fact that in the current series there were almost no cases with TLS, contrary to what is seen in DCIS and invasive cancer, where a higher number of cases with TLS is found [15]. Cases with lobular involution rarely had lymphocytes, which may reflect less immunogenicity with increasing age. In fibroadenomas, there was a range of lymphocyte infiltrate, and this seemed to correspond to the amount of reactive stroma. Those cases that had no stromal remodeling had no lymphocytes, while those that had myofibroblasts did. So, in these cases, the relationship between stromal remodeling and lymphocyte infiltration is apparent, just as is seen in DCIS and invasive cancer where cases with no stromal remodeling rarely have lymphocytes. The pattern of infiltration of lymphocytes around foci of atypical hyperplasia was similar to what is observed in DCIS, suggesting that hyperplasia seems to attract lymphocytes.

TILs stand at the nexus of the interaction between tumor and host immune response [2]. In the current study, different levels of immune cell infiltration between different lesions were apparent, suggesting a distinction between so-called developmental lesions that elicit no immune reaction, versus other benign breast lesions that do elicit an immune reaction. Also, different observations like the presence of lobulitis near zones of active stromal remodeling, due, for example, to scar tissue, similar to what is seen in DCIS and invasive breast cancer, warrant further investigation of the surrounding morphological patterns of immune infiltration in BBD, pre-invasive cancer, and invasive breast cancer. These observations also suggest that immune cells may have a developmental role in mammary organogenesis beyond a simple protective effect of the immune system.

## Conclusions

The findings from this large, nested case-control study suggest that overall infiltrating immune cell counts in normal and in BBD tissue are not associated with altered risk of subsequent invasive breast cancer. This raises the possibility that the influence of a developing tumor on the protective immune response may occur at a later stage in the natural history of breast cancer.

## Data Availability

The dataset produced for the current study is not publicly available because it was generated based on data and tissue provided by Kaiser Permanente, who retain ownership of them. Under the terms of the data use agreement established between Kaiser and Einstein in 2010, Einstein is prohibited from disclosing the data to any other party. Kaiser has agreed to release the data to bone fide investigators subject to approval by their IRB. The institutional contact person is Andrea Seykora, JD, CIP, Research Compliance Manager, Kaiser Permanente, Center for Health Research, 3800 N. Interstate Ave., Portland, OR 97227; (503) 335-6725 (office); 60-6725 (tie-line); Andrea.M.Seykora@kpchr.org.

## Abbreviations

BBD: Benign breast disease
IICC: Infiltrating immune cell count
BMI: Body mass index
TIL: Tumor-infiltrating lymphocytes
